# Supplementary material for: Impact of Catheter Ablation on Brain Microstructure and Blood Flow Alterations for Cognitive Improvements in Patients with Atrial Fibrillation: A Pilot Longitudinal Study
Source: J Clin Med. 2022 Jul 26;11(15):4346. doi: 10.3390/jcm11154346 (PMC9332426; doi:10.3390/jcm11154346)
Supplement: Supplementary file 1 [file jcm-11-04346-s001.zip › jcm-1781345-supplementary.pdf]

Supplementary Methods

Preprocessing of structural and ASL data was performed using SPM12 implemented in Matlab. Using a new segmentation algorithm [1], T1-weighted structural images obtained for each subject were segmented into six tissues including gray matter, white matter, cerebrospinal fluid, skull, soft tissue outside the brain, and air and other matter outside the head. We then used the DARTEL (diffeomorphic anatomical registration through exponentiated lie algebra) registration process to spatially normalize the six tissue probability map (TPMs) to Montreal Neurological Institute (MNI) space obtaining images with 1.5 × 1.5 × 1.5 mm^3 voxels as grey matter volume (GMV) maps. We calculated value of total brain volume for each participant by adding GM and WM in Native space for the purpose of normalization of whole GM CBF values by brain size.

Preprocessing for QUASAR-ASL images for voxel-based analyses was performed as has been fully described in previous study (Figure S1) [2–4]. For analyses of ASL measures using each individual's unnormalized segmented GM map and WM map, which were produced from the segmentation process, we created an artificial image created from the function of "1 × GM map + 2 × WM map" for each individual (we call this artificial "1 × GM + 2 × WM map" in this subsection). The contrast of this artificial "1 × GM + 2 × WM map" was similar to that of the R1 map of ASL and was used in the following preprocessing procedure of ASL images. In addition, the R1 map of ASL that is preprocessed by the abovementioned software lacked the skull and skin sections. Next, using the within-subject registration method, each individual's R1 map of ASL [which is in alignment with each individual's rCBF map was coregistered to the individual's artificial "1 × GM + 2 × WM map" together with the rCBF map. Consequently, each individual's ASL images were coregistered to each individual's T1-weighted structural image. The R1 map and rCBF map from each subject were then resliced to 1.5 × 1.5 × 1.5 mm3 voxels. Next, these coregistered ASL-related images were normalized using the normalization parameter of the T1-weighted structural image created in the abovementioned preprocessing procedure of the T1-weighted structural image.

Following this above process, using the normalized R1 map, we created a binary mask image of the areas where subjects were commonly scanned in ASL scans. The areas of this mask were defined as the areas where all the 15 participants (paired images from 8 patients with AF and single images from 7 controls) of this study had signals larger than 0 in the normalized R1 maps. We applied this common binary mask to the normalized rCBF map of each participant. This masking procedure was performed because the ASL scan cannot cover the entire brain and we had to avoid the possibility that the differences of the areas scanned affect individual differences in values of ASL measures. Normalized and masked rCBF maps were smoothed with 8 mm full-width half-maximum and acquired final QUASAR-rCBF maps for voxel-by voxel analysis.

For estimating regional cortical thickness, we analysed 3D-T1 structural images using CAT12 (Computational Anatomy Toolbox, http://www.neuro.uni-jena.de/cat/index.html) implemented by SPM12. Cortical thickness was analysed by following workflow. After preprocessing procedure as "Segment Longitudinal" pipeline, the algorithms to measure projection-based thickness use tissue segmentation to estimate white matter distance and

project the local maxima to other gray matter voxels via the neighbour relationship described by the white matter distance. Resampling surface data for cortical thickness was smoothed using a 15-mm FWHM Gaussian kernel setting.

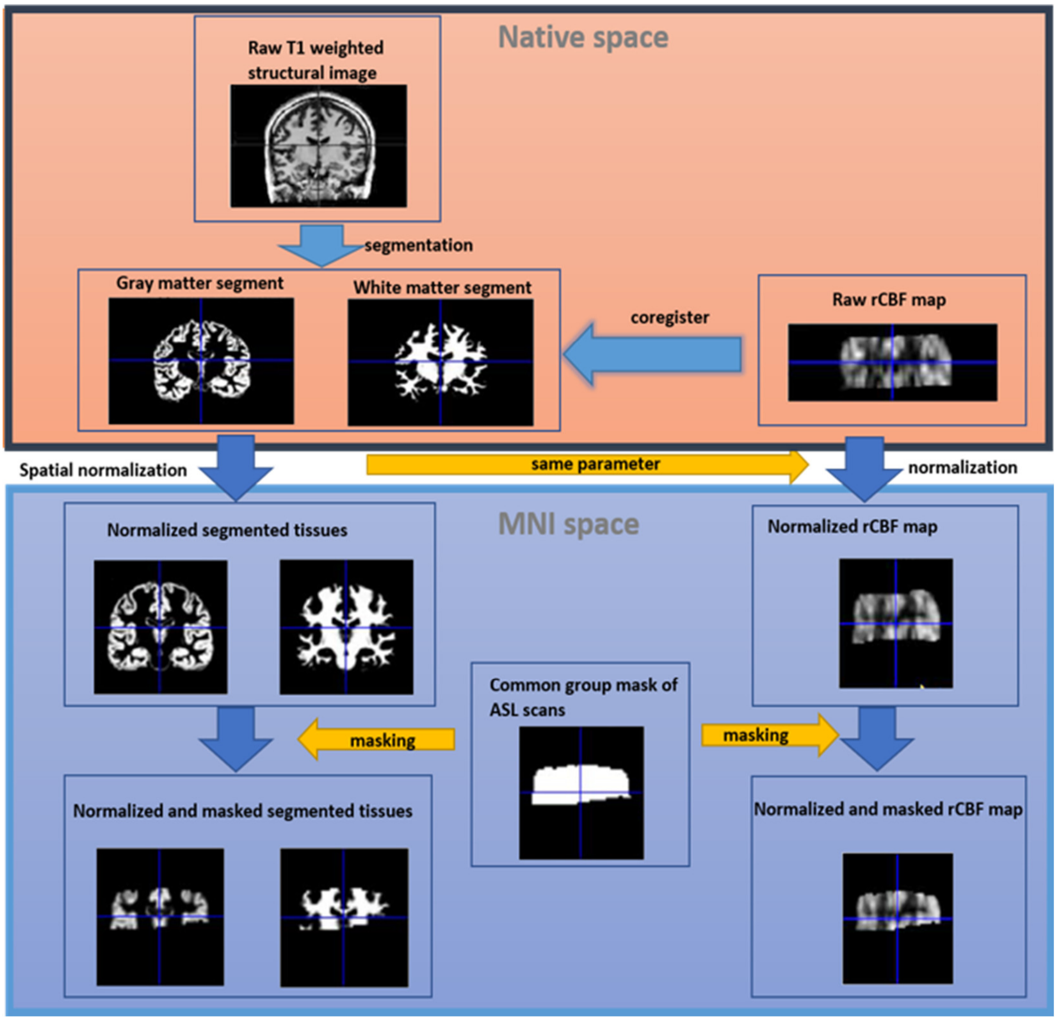

Figure S1. Overall process of structural and arterial spin labelling images for voxel-based analysis.

References

1. Ashburner, J.; Friston, K.J. Unified segmentation. *Neuroimage* **2005**, *26*, 839–851. https://doi.10.1016/j.neuroimage.2005.02.018.
2. Takeuchi, H.; Tomita, H.; Taki, Y.; Kikuchi, Y.; Ono, C.; Yu, Z.; Sekiguchi, A.; Nouchi, R.; Kotozaki, Y.; Nakagawa, S.; et al. The VEGF gene polymorphism impacts brain volume and arterial blood volume. *Hum. Brain Mapp.* **2017**, *38*, 3516–3526. https://doi.10.1002/hbm.23606.
3. Tatewaki, Y.; Higano, S.; Taki, Y.; Thyreau, B.; Murata, T.; Mugikura, S.; Ito, D.; Takase, K.; Takahashi, S. Regional reliability of quantitative signal targeting with alternating radiofrequency (STAR) labeling of arterial regions (QUASAR). *J. Neuroimaging* **2014**, *24*, 554–561. https://doi.10.1111/jon.12076.
4. Tatewaki, Y.; Mutoh, T.; Sato, H.; Enoki, R.; Kobayashi, A.; Muranaka, M.; Yamamoto, S.; Yagi, T.; Taki, Y. Catheter ablation improves QUASAR-arterial spin labeling based regional brain perfusion in the posterior cingulate cortex and auditory-verbal memory function in elderly patients with atrial fibrillation. *Circulation* **2020**, *142* (Suppl S3), A13806. https://doi.10.1161/circ.142.suppl_3.13806.
